# Craniofacial microsomia – more than a structural malformation

**DOI:** 10.1111/ocr.12592

**Published:** 2022-06-20

**Authors:** Louhi Kuu‐Karkku, Auli Suominen, Anna‐Liisa Svedström‐Oristo

**Affiliations:** ^1^ Pediatric Dentistry and Orthodontics, Institute of Dentistry University of Turku Turku Finland; ^2^ Department of Community Dentistry, Institute of Dentistry University of Turku Turku Finland

**Keywords:** congenital deformity, craniofacial microsomia, hospital districts, specialized healthcare services, structural malformation

## Abstract

**Objectives:**

To analyse the prevalence and distribution of craniofacial microsomia (CFM) cases in Finland and their most frequent comorbidities. The second aim was to analyse the patients' need for specialized healthcare services.

**Materials and methods:**

Data were gathered from two complementary registers: The Register of Congenital Malformations and the Care Register for Social Welfare and Health Care (Hilmo) of the Finnish Institute for Health and Welfare (THL).

**Results:**

The prevalence of CFM patients in Finland was 1:10 057. They were evenly distributed across the five university hospital districts. Their most frequently used ICD‐10 diagnosis codes were F40‐48 (Neurotic, stress‐related and somatoform disorders), 60% of patients in adolescent and adult psychiatry; Q67.0 (Facial asymmetry), 43% in plastic surgery; Z00.4 (General psychiatric examination, not elsewhere classified), 31% in child psychiatry; Z31.5 (Genetic counselling), 28% in clinical genetics and Q67.40 (Other congenital deformities of the skull, face and jaw, Hemifacial atrophy), 18% in dental, oral and maxillofacial diseases. Of the patients, 70% had had visits in clinical genetics, 60% in plastic surgery, 41% in dental, oral and maxillofacial diseases, 28% in adolescent/adult psychiatry and 21% in child psychiatry. The majority of the patients' plastic surgery visits were concentrated in one university hospital. Other services were mainly provided by patients' own hospital districts.

**Conclusions:**

Even though the majority of CFM patients' visits in specialized healthcare services are related to correction of facial asymmetry and ear malformations, the obvious need for psychiatric care was apparent in all age groups.

## 1 INTRODUCTION

Craniofacial microsomia (CFM) is the second most common congenital facial deformity after cleft lip and cleft palate. It is a developmental disorder in structures originating from the first and second pharyngeal arches during the 4th and 6th weeks of pregnancy. Its distinctive features are underdevelopment of the mandible and/or maxilla, ear, orbit, facial soft tissue and facial nerve. CFM occurs most often on the right side of the face. In approximately 10% of cases, the disorder occurs bilaterally. Depending on the severity, CFM can cause problems in nutrition intake, breathing, hearing and facial muscle function. Furthermore, it can be related to epibulbar dermoids, vertebral malformations, cardiovascular malformations and malformations in lungs, brain and genitourinary system.^1^, ^2^, ^3^, ^4^, ^5^ Fan et al reported that 10% of CFM cases had cleft lip/palate and 23% macrostomia.^4^ The terminology used for craniofacial microsomia varies and other terms are also used such as hemifacial microsomia (HFM), oculo‐auriculo‐vertebral dysplasia (OAVS) or Goldenhar syndrome.^2^, ^6^

Estimates of the prevalence of CFM range from 1:3500 to 1:45 000.^5^ The aetiology of the disorder is unclear, but both genetic and environmental factors, for example, mother’s exposure to certain teratogens (such as thalidomide, retinoic acid, primidone and cocaine) during pregnancy have been suggested.

According to one theory, the causal factor might be an early disturbance in the circulatory system. In 1973, Poswillo^7^ studied this theory in mice: During the 6th week of gestation, an artificial disruption in stapedial artery blood flow resulted in similar facial disorders as seen in CFM patients. Other, more recently suggested risk factors for CFM include maternal smoking during the second trimester of pregnancy, maternal diabetes and multiple births.^1^, ^2^, ^3^

Although most CFM cases are sporadic, it also occurs in families. In these cases, the autosomal inheritance can manifest either dominantly or recessively. The diagnostic challenges in CFM are caused by the wide spectrum of symptoms and the lack of common diagnostic criteria. Pruzansky’s well‐known classification for CFM (1969) describes the severity of mandibular deformity, while SAT‐, OMENS‐ and OMENS Plus‐classifications include also other developmental disorders of the face and soft tissues. With regard to differential diagnostics, CFM includes similar symptoms as, for example, CHARGE, Townes‐Brocks and Treacher‐Collins syndromes.^1^, ^2^, ^3^, ^8^, ^9^, ^10^, ^11^ At the end of 2020, a Working Group of Craniofacial Microsomia published a consensus article focusing on uniform criteria for CFM in Europe.^12^

According to a decree of the Ministry of Social Affairs and Health, the diagnostics and treatment of CFM in Finland are carried out in university hospitals. In CFM cases that include cleft lip and/or palate, the entire treatment protocol is planned and early‐stage surgeries are implemented at Helsinki University Hospital, or in cooperation with the Oulu University Hospital.^13^, ^14^ For each patient, multidisciplinary treatments and their timing are planned individually, for example, with regard to the schedule of craniofacial growth.

An ear malformation, characteristic of CFM, may range from a small fibroma in front of the ear to microtia or total absence of the auricle. The malformed ear and other soft tissue deficiencies can be repaired with plastic surgery.^6^ Ear malformation may also be associated with structural deviations in the auditory ossicles, resulting in hearing loss or deafness. While hearing loss has a huge impact on everyday life, hearing should be monitored repeatedly throughout the growth period.^2^, ^15^ Given the increased risk of psychosocial problems during childhood, it is important to provide children with CFM with psychosocial support early on.^15^, ^16^, ^17^, ^18^ Nonetheless, differences in experienced quality of life appear in childhood to be small compared to healthy peers.^19^

Further, effective monitoring is also needed, because a small, underdeveloped mandible may constrict the respiratory tract and lead to obstructive sleep apnoea (OSA).^2^ In the most severe developmental deficiencies, with completely missing condylar cartilage, reconstruction of the mandible may be done with a costochondral graft. Distraction osteogenesis and costochondral grafts early in the growth phase are justified especially when a child with CFM has difficulties breathing or eating.^6^ Orthodontic‐surgical corrections of severe developmental jaw discrepancies can be made only after growth has ceased. In fact, CFM patients treated during the growth phase may later require a new orthodontic‐surgical treatment period once they have reached full growth.^6^ For a more detailed description of early treatment of clefts, ear problems and OSA, see, for example, the review by Hopkins et al^20^

In order to effectively arrange public healthcare services that meet the needs of a special group of patients, it is vital to be familiar with the prevalence and distribution of these cases as well as the associated resource needs. This study aimed at analysing (1) the prevalence and distribution of CFM cases in Finland, (2) the prevalence of other, most common comorbidities and (3) the use of specialized healthcare services related to these diagnoses.

## 2 MATERIALS AND METHODS

This register study is based on data from two complementary registers: the Register of Congenital Malformations and the Care Register for Health Care (HILMO) of the Finnish Institute for Health and Welfare (THL). The data included in these registers are gathered widely from the Finnish health care authorities, institutions and professionals (e.g. Medical Birth Register, physicians, hospitals, prenatal and child welfare clinics). The Care Register for Health Care includes information on outpatient services in specialized health care. All documentations considering congenital malformations are based on verbal diagnosis, ICD‐codes, descriptions of the confirmed or suspected aetiology, pattern of anomaly and time and diagnostic method of detecting the anomaly. These data are complemented by x‐rays and other examinations. During 1988‐2013, the data covered 156 live‐birth CFM patients reported to the registers. All patients were diagnosed with craniofacial microsomia either as a single developmental disorder or in combination with a cleft lip and/or palate (ICD‐10 codes Q35‐37).^21^ Patients' health records were collected between 1996 and 2013 and included demographic data (year and place of birth, municipality of residence, gender) and ICD‐10 diagnosis codes, visits to specialized medical care and, since 2011, also primary health care visits. During 1988‐2013, the hospital district and its specific catchment area responsible for specialized medical care were determined on the basis of patients' municipality of residence. Students whose studies have lasted for more than 1 year, have, since 1994, had the option to change their domicile to the municipality where they study.^22^

The distribution of ICD‐10 diagnosis codes was examined in the following nine specialties: adolescent psychiatry, adult psychiatry, plastic surgery, child psychiatry, clinical genetics, dental, oral and maxillofacial diseases, clinical dental care, orthodontics and oral and maxillofacial surgery. For each patient, each ICD‐10 diagnosis code was considered only once (either as a primary or secondary diagnosis) in each specialty. The number of visits without any ICD‐10 diagnosis code was considered in assessment of the treatment burden in each specialty.

The prevalence of CFM cases and CFM patients' visits in different clinics were examined nationwide by numbers and percentages. Gender differences and distributions of the visits between hospital districts were analysed with the Kruskal–Wallis test (IBM SPSS Statistics, Version 25). *P*‐values of <0.05 were interpreted as statistically significant.

## 3 RESULTS

Between genders or hospital districts, no statistically significant differences were found in the occurrence of CFM or in the number of visits per specialty. Detailed data on the distribution of the subjects and their visits in specialized health care are presented in Tables 1 and 2.

**TABLE 1:** Distribution of 1988‐2013 born CFM patients and their visits by specialty between hospital district A‐E catchment areas. Comparisons made with Kruskal–Wallis test

Subjects	*N*	A *n*	B *n*	C *n*	D *n*	E *n*	*P*‐value
Diagnosed CFM cases	156	18	28	18	26	66	0.479
Male	78	10	14	7	13	34	
Female	78	8	14	11	13	32	0.750
Visits by specialty	Number of visits	A %	B %	C %	D %	E %	
Adolescent psychiatry^a^	12	17.0	8.0	0.0	17.0	58.0	0.506
Adult psychiatry^b^	8	25.0	0.0	0.0	37.5	37.5	0.752
Child psychiatry	32	19.0	15.0	3.0	22.0	41.0	0.173
Clinical genetics, (<18 years old)	55	13.0	20.0	13.0	3.0	51.0	0.959
Clinical genetics (≥18 years old)	8	37.5	37.5	0.0	0.0	25.0	0.267
Plastic surgery	94	10.0	16.0	4.0	15.0	55.0	0.403
Dental, Oral and Maxillo‐facial diseases^c^	64	14.0	30.0	17.0	33.0	6.0	0.552
Dental, Oral and Maxillofacial diseases	59	15.0	27.0	17.0	36.0	5.0	0.768
Oral and Maxillofacial surgery	13	8.0	0.0	38.5	38.5	15.0	0.434
Orthodontics	15	0.0	73.0	7.0	13.0	7.0	0.412
Clinical dental care	10	10.0	10.0	60.0	10.0	10.0	0.296

^a^ Adolescent psychiatry: 13‐17 years old (born in 1996‐2000).

^b^ Adult psychiatry: ≥ 18 years old (born in 1988‐1995).

^c^ Includes 33 patients with overlapping visits in the specialties of Oral and Maxillofacial Diseases, Oral and Maxillofacial Surgery, Orthodontics and Clinical Dental Care.

**TABLE 2:** Descriptive data on CFM patients' visits in specialized health care by specialty

Speciality	Number of all subjects	Number of patients	Percentages %	Number of all visits	Mean of visits/patient	Median of all visits	Range of visits
Adolescent psychiatry^a^	83	12	14	261	21.8	9.5	2‐83
Adult psychiatry^b^	58	8	14	131	17.2	4.0	2‐93
Child psychiatry	156	32	21	492	15.4	9.5	1‐114
Clinical genetics^b^	98	55	56	93	1.7	2.0	1‐4
Clinical genetics^c^	58	8	14	11	1.4	1.0	1‐2
Plastic surgery	156	94	60	1170	12.5	6.5	1‐63
Dental, Oral and Maxillofacial diseases^d^	156	64	41	1106	17.3	7.5	1‐91
Dental, Oral and Maxillofacial diseases	156	59	38	924	15.7	5.0	1‐91
Oral and Maxillofacial surgery	156	13	8	49	3.8	2.0	1‐15
Orthodontics	156	15	10	84	5.6	4.0	1‐18
Clinical dental care	156	10	6	49	4.9	2.0	1‐33

^a^ 13‐17 years old (born in 1996‐2000).

^b^ <18 years old (born in 1996‐2013).

^c^ Includes 33 patients with overlapping visits in the specialties of Oral and Maxillofacial Diseases, Oral and Maxillofacial Surgery, Orthodontics and Clinical Dental Care.

^d^ ≥18 years old (born in 1988‐1995).

The most common ICD‐10 diagnosis codes by specialty were the following:

*Adolescent psychiatry and adult psychiatry:* F40‐48 (Neurotic, stress‐related and somatoform disorders) in 60%, F30‐34 and F38‐39 (Mood [affective] disorders) in 50% of patients. ICD‐codes were not reported in 39% of visits.
*Plastic surgery:* Q67.0 (Facial asymmetry) in 43%, Q17.2 (Microtia) in 39%, Q16.0 (Congenital absence of [ear] auricle) in 26% and Q35.1 Cleft hard palate in 14% of patients. ICD‐codes were not reported in 2% of visits.
*Child psychiatry:* Z00.4 (General psychiatric examination, not elsewhere classified) in 31% and Z63.7 (Other stressful life events affecting family and household) in 22% of patients. ICD‐codes were not reported in 41% of visits.
*Clinical genetics:* Z31.5 (Genetic counselling) in 28% and Q67.40 (Other congenital deformities of the skull, face and jaw, Hemifacial atrophy) in 6.3% of patients. ICD‐codes were not reported in 8% of visits.
*Dental, oral and maxillofacial diseases:* Q67.40 (Other congenital deformities of the skull, face and jaw, Hemifacial atrophy) in 18% and K07.10 (Anomalies of jaw‐cranial base relationship, Asymmetry of jaw) in 16% of patients. ICD‐codes were not reported in 10% of visits.

Visits related to clinical genetics and psychiatry occurred mainly in patients' own hospital districts; the same was true of visits related to dental, oral and maxillofacial diseases. For two patients, some of the visits in the latter specialty were handled in another hospital district. The majority of plastic surgery visits were concentrated in one university hospital. Two of the four university hospitals referred there all, and two others, 50%‐67% of the cases, while one in three cases was treated in cooperation.

## 4 DISCUSSION

According to Statistics Finland, there were 1 568 913 live births in Finland between 1988 and 2013. During this period, a total of 156 live‐birth CFM cases were registered, corresponding to a prevalence of 1:10 057. This is well in line with previous estimates of the prevalence of CFM.^23^

Most of the patients' visits to specialized medical care were related to plastic surgery, followed by dental, oral and maxillofacial surgery (including orthodontics) and psychiatry. According to Sourander et al,^24^ about 7% of Finnish children and adolescents have sought psychiatric help between the ages of 8 and 16. In contrast, approximately 1 in 5 CFM patients needed psychiatric support in childhood. Among adolescents and adults, the need was slightly lower, 14%. As the percentages of children’s, adolescents’ and adults’ psychiatric visits remained fairly stable, the patient’s age did not appear to affect the need for psychiatric support. However, it should be noted that the Care Register for Social Welfare and Health Care (HILMO) was expanded to include data on outpatient visits (the so‐called AvoHILMO) first in 2011. During 1996‐2010, the data include only visits to specialized medical care. In 1996‐1998, the data included only those specialized medical care visits requiring care in inpatient care, and until 2010, included only visits to specialized medical care. As psychiatric visits may have been carried out in primary health care as well, the current data underestimate the real number of psychiatric visits.

The second most common ICD‐code for psychiatric visits in children (Z63.7, Other stressful life events affecting family and household) refers to the targeting of psychiatric support to the whole family. According to a previous study, parents' worry about their CFM‐affected child’s survival has been even greater than the impact of CFM on the child’s quality of life.^19^ However, although children and adolescents with CFM feel that they are doing better than their parents expect, they also face bullying.^15^, ^25^ Therefore, it is more than vital to recognize the need for psychological support from early on.

Visits to the clinical genetics specialty provide families with a CFM‐affected child with support and information about the developmental disorder. Somewhat surprisingly, only slightly more than one in two children and adolescents with CFM under 18 years of age had had such visits. It is possible that the visits had not been offered routinely; it is also possible that families had not prioritized these visits in relation to various other treatment procedures. Given that the adults included in the current study were young, only 18‐25 years old, the low participation in genetic counselling may be understandable. However, in a recent article, Birgfeld and Heike^2^ recommend that a genetic counselling is provided for each CFM patient in both childhood and adulthood.

With regard to the structures affected by the developmental disorder, the need for plastic surgery among CFM patients is obvious. In the current study, almost two in three patients needed plastic surgery, and their number of visits related to ear repair surgeries varied from 1 to 63. A recent study by Hamilton et al,^15^ emphasized the importance of involving patients in decision making regarding ear reconstruction. Indisputably, it would be advisable to listen to patients' wishes and worries whenever possible.

In Finland, the ICD‐9 codes were implemented in 1987. In order to get as structured data as possible, collection of the study material started from 1988. At completion of the data gathering, the two applied registers were first available until the end of 2013. Thus, the materials of the current study include all live‐born subjects with a CFM diagnosis during 1988‐2013 who were registered in the applied THL databases. The wide coverage spanning the whole country and a period of 25 years can be considered strengths of this study.

However, there are limitations as well. Although the list of ICD‐10 diagnosis codes in Finland was ready for use in 1992, the codes were implemented only in 1996.^26^ Prior to that, application of ICD‐10 diagnosis codes had not been a common practice and all markings had been written manually on patient records. Presumably due to the tight implementation time, a share of ICD‐10 diagnosis codes were missing from the registers. However, all visits – with or without an ICD‐10 diagnosis code – were included in assessments of treatment load in each specialty.

As stated, there was variation also in recording practices with regards to time periods and treatment units. In spite of missing health records in childhood, the older age groups were included in the material, because their data add important information on the need of specialized health care at a later age. However, to some degree, the data underestimate the need for various resources reflecting more or less the minimum requirements. Therefore, the results should be interpreted with caution.

Annually, only a few CFM patients are born in Finland. Given that the majority of visits related to plastic surgery are provided by one university hospital, other specialized healthcare services are provided by patients' own hospital districts. Today, every patient can freely select the place of treatment, and referrals from one place to another can be done according to need.^27^

## 5 CONCLUSIONS

- In Finland, the prevalence of live‐birth CFM cases was about 1:10 000 during 1988‐2013.
- Treatment of CFM is multidisciplinary. Although most of the patients' visits to specialized medical care are related to functional and aesthetic corrections, the psychological burden of CFM affects patients in all age groups.
- Due to the lack of ICD‐10 diagnosis codes and variation in recording practices, it is obvious that the actual need for various treatments exceeds the numbers presented in this study.

## AUTHORS' CONTRIBUTIONS

Design of the study: Louhi Kuu‐Karkku and Anna‐Liisa Svedström‐Oristo. Statistical planning: Auli Suominen. Statistical analyses: Louhi Kuu‐Karkku and Auli Suominen. Writing (Original draft preparation): Louhi Kuu‐Karkku, Auli Suominen, Anna‐Liisa Svedström‐Oristo.

## CONFLICT OF INTEREST

The authors report no conflict of interest.

## Data Availability

Research data are not shared.
